# Mass Spectrometry for Neurobiomarker Discovery: The Relevance of Post-Translational Modifications

**DOI:** 10.3390/cells11081279

**Published:** 2022-04-09

**Authors:** Rita Azevedo, Chloé Jacquemin, Nicolas Villain, François Fenaille, Foudil Lamari, François Becher

**Affiliations:** 1 CEA, INRAE, Département Médicaments et Technologies pour la Santé (DMTS), SPI, Université Paris-Saclay, 91191 Gif-sur-Yvette, France; chloe.jacquemin@cea.fr (C.J.); nicolas.villain@cea.fr (N.V.); francois.fenaille@cea.fr (F.F.); 2 Institut du Cerveau (ICM), Pitié-Salpêtrière Hospital, 75013 Paris, France; 3 Department of Neurology, Institute of Memory and Alzheimer’s Disease, Pitié-Salpêtrière Hospital, AP-HP Sorbonne Université, CEDEX 13, 75651 Paris, France; 4 Department of Metabolic Biochemistry (AP-HP Sorbonne), Pitié-Salpêtrière Hospital, CEDEX 13, 75651 Paris, France; foudil.lamari@aphp.fr

**Keywords:** biomarkers, blood, brain, cerebrospinal fluid, crosstalk, mass spectrometry, neurodegenerative diseases, post-translational modifications, proteoforms

## Abstract

Neurodegenerative diseases are incurable, heterogeneous, and age-dependent disorders that challenge modern medicine. A deeper understanding of the pathogenesis underlying neurodegenerative diseases is necessary to solve the unmet need for new diagnostic biomarkers and disease-modifying therapy and reduce these diseases’ burden. Specifically, post-translational modifications (PTMs) play a significant role in neurodegeneration. Due to its proximity to the brain parenchyma, cerebrospinal fluid (CSF) has long been used as an indirect way to measure changes in the brain. Mass spectrometry (MS) analysis in neurodegenerative diseases focusing on PTMs and in the context of biomarker discovery has improved and opened venues for analyzing more complex matrices such as brain tissue and blood. Notably, phosphorylated tau protein, truncated α-synuclein, APP and TDP-43, and many other modifications were extensively characterized by MS. Great potential is underlying specific pathological PTM-signatures for clinical application. This review focuses on PTM-modified proteins involved in neurodegenerative diseases and highlights the most important and recent breakthroughs in MS-based biomarker discovery.

## 1. Introduction

Neurodegenerative diseases are age-dependent disorders characterized by the progressive degeneration of neural cells, affecting nearly 57 million people worldwide and 10 million new cases every year [1]. These diseases impose substantial medical and public health burdens [1]. They are increasingly prevalent and incidental, mainly due to an increase in life expectancy, and are expected to increase dramatically in the near future [1]. The etiological factors of these diseases are poorly understood and result from genetic and environmental factors [2,3]. Mostly in high-income countries, diagnosis can combine clinical examination, including neuropsychological testing, brain imaging (e.g., 18F-fluorodeoxyglucose positron emission tomography (PET), magnetic resonance imaging, and amyloid or tau PET) [4,5], and cerebrospinal fluid (CSF) biomarkers in the case of Alzheimer’s disease (AD) [6]. Particularly, neuroimaging associated with CSF biomarkers was introduced to increase the diagnostic accuracy of AD, especially at the early stage and in the case of atypical clinical presentation [7]. Currently performed on CSF, most clinical laboratory tests combine elevated levels of total and phosphorylated tau proteins (e.g., pT181 and pT217) and reduced soluble amyloid-beta peptide (Aβ)42 levels or Aβ42/40 ratios allow distinguishing AD patients from non-AD individuals with a mean accuracy of 85–89% [8,9]. Such biomarkers are currently unavailable for non-AD neurodegenerative diseases. Besides, no validated blood-, saliva- or urine-based biomarkers and no specific therapy is currently available for clinical use in neurodegenerative diseases [4,5]. According to the anatomical tropism of the neurodegenerative processes, i.e., the specifically affected brain tissue areas, some of these diseases start with cognitive or behavioral impairments (dementia), while others first begin with movement disorders, sensory-motor deficits, or epilepsy [10,11,12,13]. The broad spectrum of primary neurodegenerative disorders mainly includes AD, frontotemporal lobar degeneration (FTLD), dementia with Lewy bodies (DLB), Parkinson’s disease (PD), multiple system atrophy (MSA), progressive supranuclear palsy (PSP), corticobasal degeneration (CBD), Creutzfeldt–Jakob disease (CJD), amyotrophic lateral sclerosis (ALS), and Huntington’s disease (HD) [14]. There is a substantial overlap in the clinical symptoms of these diseases, which complicates an effective and accurate diagnosis, particularly in the early stages [4,15]. Besides, co-occurring pathologies are frequent and further blur the clinical phenotype boundaries, representing a challenge for identifying specific biomarkers [16].

While abnormal protein aggregates typically define neurodegenerative diseases, it is unclear whether these abnormalities are driving the disease or are themselves consequences of other underlying processes [17]. Several fundamental processes are associated with progressive neuronal dysfunction and death, including abnormal protein dynamics, proteotoxic stress, dysfunctions in the ubiquitin-proteasome and autophagosomal/lysosomal systems, oxidative stress, programmed cell death, and neuroinflammation [18]. Abnormal protein aggregates can take several histopathological forms in the brain tissue: neurofibrillary tangles, Pick’s bodies, tufted astrocytes, Lewy bodies, amyloid plaques, among others [18]. For instance, AD is a dual proteinopathy characterized by accumulating tau aggregates in neurofibrillary tangles and extracellular aggregates of Aβ plaques [19,20]. The aggregation of α-synuclein into Lewy bodies and neurites is characteristic of synucleinopathies, including PD and DLB, while glial cytoplasmic inclusions of α-synuclein in oligodendrocytes can occur in MSA [21,22]. The accumulation of tau 4R isoforms, i.e., isoforms that contain four carboxy-terminal repeat domains, in neuronal and glial cells is the main characteristic of PSP and CBD. The transactive response DNA binding protein-43 (TDP-43) is the main component of intracellular ubiquitin inclusion bodies in pathological deposits in ALS and FTLD [12,18]. Several challenges, including the complexity of protein aggregation, misfolding, pathology propagation processes, and the immune response, need to be explored for successful translational research on neurodegenerative disorders.

The discovery of biomarkers in neurodegenerative diseases remains an important challenge in modern medicine [17]. An ideal biomarker should reflect pathological changes in the brain with accurate performance, differentiating forms of neurodegenerative diseases [23]. Currently, neurodegenerative disease research for biomarker discovery is focused on: (1) early disease biomarkers, present in both brain tissue and biological fluids (e.g., CSF and blood) [8,9,23,24,25]; (2) detection of molecular signatures, i.e., the combination of proteins or proteoforms (more details about proteoforms can be found elsewhere [26]), that correlate with the neuropathological processes or disease status (e.g., rate of progression, treatment response) [23,27,28,29]; and, (3) surrogate biomarkers for developing disease-modifying therapies [30,31,32,33,34].

Mass spectrometry (MS) is characterized by high detection sensitivity, specificity, and multiplexing capability for biomarker discovery and validation [35]. MS-based proteomics has considerably extended our knowledge about the occurrence and dynamics of protein post-translational modifications (PTMs). Profiling the main protein players of neurodegeneration in the brain using MS can provide new insights into the role of aberrant PTM patterns in the pathological process and identify signatures with potential diagnostic or therapeutic relevance. This review focuses on recent developments in MS-based analysis of PTM-modified proteins, emphasizing the most prevalent neurodegenerative diseases and providing an integrated perspective on the future directions in the biomarker discovery field.

## 2. Mass Spectrometry for Neurobiomarker Discovery

### 2.1. Proteomics with Mass Spectrometry

Proteomics is a core technology in current post-genomic system biology approaches to understand molecular mechanisms underlying normal and disease phenotypes. In its present state, the current revolution in proteomics and systems biology relies on decades of technological, conceptual, and instrumental developments for fast and sensitive new analytical tools. A notable methodological advance was the discovery and development of protein soft desorption ionization, as recognized by the 2002 Nobel Prize in Chemistry [36]. Accordingly, several high-throughput technologies have been developed to investigate proteomes in depth. The most commonly applied are MS-based and gel-based techniques (e.g., two-dimensional gel electrophoresis) [37,38].

Using MS instruments, proteins can be studied using a top-down approach by analyzing intact proteins or a bottom-up approach by measuring proteolytically generated peptides. The bottom-up approach is the most mature and widely used proteomic approach for protein identification, PTM discovery, and quantification. The MS-based proteomics workflow consists of ionization of a molecule in an ionization source, such as electrospray ionization (ESI) or matrix-assisted laser desorption ionization (MALDI), and separation of the ionized species according to their mass-to-charge ratio in a mass analyzer (ion trap, quadrupole, or high-resolution time-of-flight (TOF), and more recently Orbitrap mass analyzers) [39]. Tandem mass spectrometers have the additional capability of selecting and fragmenting a precursor ion to obtain structural information (MS/MS) [39]. This basic process is the foundation for the three common variants of bottom-up proteomics workflows: data-dependent acquisition (DDA), data-independent acquisition (DIA), and targeted data acquisition (tMS2). In DDA, a defined number of precursor ions from the full scan are selected based on their respective intensity or charge. In DIA, the full MS spectrum is acquired, followed by a series of sequential MS/MS spectra of predefined isolation windows that subdivide a larger *m*/*z* region (e.g., sequential window acquisition of all theoretical fragment ions (SWATH)) [40]. In tMS2, a list of precursors is selected for fragmentation, followed by the detection of a few (typically three product ions; selected/multiple reaction monitoring (SRM/MRM)) or most (parallel reaction monitoring, PRM) major product ions [41]. Fragmentation approaches within the MS include approaches relying on the collision of ions with inert gases (e.g., collision-induced dissociation (CID)) and higher energy transfer methods (e.g., electron capture dissociation (ECD), electron-transfer dissociation (ETD)) [42,43]. These fragmentation techniques have emerged to generate information-rich spectra to identify and localize PTMs.

Quantitative MS-based methods to analyze protein abundance and PTMs have evolved over the last 10–20 years of technological advancements in mass spectrometer design and the development of various label-based and label-free-based methods to allow for quantitative analysis to detect changes in protein abundance across multiple biological and technical replicates. In-depth MS-based approaches, including both label (isotope-coded affinity tag (ICAT), isobaric tagging for relative and absolute quantitation (iTRAQ), tandem mass tags (TMT), stable isotopic labeling by amino acids in cell culture (SILAC), stable isotope-labeled compounds (SILAM)), label-free (peptide spectral counts, extracted ion intensity), and absolute quantification methods [44,45], are frequently being applied to neuroscience. One of the deepest human-brain proteomes was generated by a quantitative proteomic analysis of 80 post-mortem human brain tissues through tandem mass tag (TMT) isobaric labeling and synchronous precursor selection-based MS3 (SPS-MS3, widely accepted for improved multiplex quantitation accuracy) on an Orbitrap Fusion Tribrid mass spectrometer [30]. Tissues being studied were from two brain regions (frontal cortex and anterior cingulate gyrus) of AD, PD, co-morbid AD/PD, and healthy controls. This study identified 11,840 protein groups representing 10,230 coding gene products, which map to ~65% of the protein-coding genes in the brain when overlapped with available brain-specific (cerebral cortex) RNA-seq data [30].

Today, proteomics remains a multidimensional, rapidly evolving, and open-ended endeavor. Proteomic datasets are beginning to challenge the depth and breadth of transcriptomic datasets, thanks to advances in multiplex labeling technologies, offline fractionation, and high-resolution MS [30,46]. Apart from differential protein expression, MS-based proteomics can be combined with up-front enrichment of organelles or other cell compartments or immunoprecipitation of one or more proteins of interest [47]. For example, MS-based workflows can be coupled with laser capture microdissection (LCM), magnetic-activated cell sorting, or fluorescence-activated cell sorting (FACS) for obtaining subpopulations of cells (or very small regions of interest in the case of LCM) [48]. These approaches are very powerful but underused [48]. Similarly, an enrichment step for peptides bearing specific PTMs allows for system-wide analysis of these modifications, with phosphoproteomics being a good example [49]. Of course, MS-based proteomics is only made possible by the availability and development of gene and genome sequence databases and bioinformatic tools [50].

### 2.2. Determination of Post-Translational Modifications Using Mass Spectrometry

Post-translational modifications of proteins modulate their molecular function and the spatial-temporal distribution in cells and tissues [42]. Specifically, protein modifications comprise essential mechanisms that eukaryotic cells use to diversify their protein functions and dynamically coordinate their signaling networks. A large class of PTMs is represented by chemical moieties covalently attached to proteins by various enzymes. These PTMs include phosphorylation (+79.966 Da), acetylation (+42.010 Da), methylation (+14.016 Da), ubiquitination (+383.228 Da), sulfation (+79.957 Da), SUMOylation (+600.250 Da and +599.266 Da), or glycosylation (>203 Da) (a more comprehensive list of PTMs can be found elsewhere [50,51,52,53]). Proteins can also be subject to proteolytic cleavage(s), de. PTMs reversibly or irreversibly alter the structure and properties of proteins through biochemical reactions. As many as 300 PTMs of proteins are known to occur physiologically [39,52].

Interestingly, a protein sequence can have many different modifiable amino acids, but not all will be modified simultaneously or within the same copy of that protein. Competition can exist within a specific residue between various modifications [54]. Overall, intra- and inter-protein PTM crosstalks provide the necessary nano-switches and recognition motifs for the rapid cell implementation of signaling networks. Precisely, the fundamental role of PTMs as nano-switches of cellular homeostasis controls the flow of information through a particular protein, helping to shape dynamic biological processes, including genetic silencing, cellular growth, differentiation, and apoptosis. Therefore, they act both as drivers and markers for pathogenesis and, thus, aberrant PTM patterns have been associated with numerous diseases such as cancer [55], diabetes [56], and autoimmunity [57]. So, as analytical approaches for mapping and quantifying PTMs have undergone impressive progress and are now routinely analyzed (e.g., CSF phosphorylated tau for AD diagnosis), much biomedical research on understanding the role of PTMs in cellular communications has been conducted [42,58]. For example, global changes in histone PTM abundances are quantifiable with nearly routine proteomics analyses, and it is now possible to determine combinatorial patterns of modifications [58].

Determination of PTM profiles of proteins is fundamental in elucidating the intricate processes that govern cellular events under normal and pathological conditions. Still, many PTMs lack a clear understanding of their regulation or role in biology. Conventionally, PTM-modified amino acids have been identified by Edman degradation (but PTMs can be unstable under this technique, e.g., phosphorylation), amino acid analysis, isotopic labeling, or immunochemistry. Traditional protein-detection methods such as enzyme-linked immunosorbent assay (ELISA) and western blots are essential in PTMs identification and/or quantification. Nevertheless, the disadvantages of conventional protein-detection methods include: (1) antibodies can be non-specific, with possible cross-reactions, and expensive, mainly when complex matrices are analyzed; and (2) antibody-based methods rely on antibody availability and capacity to downscale to the trace level of the modified peptide. On the other hand, standard mass spectrometers have high speed (scan rate >10 Hz), high resolution (>60,000 resolving power), capacity to identify and quantify PTMs (simultaneously and with information about PTM site localization) in complex mixtures of proteins, and the ability to discover novel PTMs. In addition, the possibility to combine MS acquisition with online nano-liquid chromatography, which led to highly sensitive analyses (<fmol), has made MS the technique of choice for PTMs discovery and quantification [59].

Although mass spectrometers have experienced a rapid advance in past decades, mapping of PTMs in proteomics using MS is a demanding task because most PTMs are: (i) low abundant; (ii) substoichiometric; (iii) labile during MS and MS/MS analysis; (iv) variable according to peptide hydrophobicity, which complicates PTM sample handling and purification before MS; (v) may affect the cleavage efficiency of proteases, such as trypsin, to generate miscleaved or large peptide products; (vi) may reduce the ionization and detection efficiency (and therefore the sensitivity); and, (vii) multisite PTMs may generate very complicated MS and MS/MS data sets that are difficult to interpret. PTM analysis from complex protein digests in a standard LC-MS/MS analysis requires highly specialized and sensitive PTM-specific enrichment methods. Peptides must be specifically enriched to deal with the coexistence of a large number of unmodified peptides, which severely suppresses the detection of PTM peptides to achieve large-scale analysis. Numerous PTMs currently lack or have inadequate platforms for selective enrichment, making proteomic studies rely on fractionation to identify the PTM of interest. Nevertheless, very well implemented methods exist for the enrichment of the most common PTMs, including immunoprecipitation, affinity chromatography (e.g., titanium dioxide (TiO_2_), immobilized metal affinity chromatography (IMAC), lectins), and chemical derivatization [60]. With the ongoing advances in instrumentation, methods, and bioinformatics, the number of known PTMs is likely to increase.

### 2.3. PTM-Focused Neuroproteomics: Relevance and Challenges

Neuroproteomics has focused on understanding protein-driven molecular mechanisms during a disease by studying proteins that can be used as novel biomarkers to facilitate diagnosis, monitoring, and prognosis [61]. For instance, it is known that changes in protein abundance, protein turnover, and protein function (as also in PTMs) can lead to local changes in neuronal function [61,62]. Since most neurodegenerative disorders have specific neuroanatomical tropism, neuroproteomics of the affected tissue and its surroundings during disease development is critical for understanding the complexity of these diseases.

More than two decades have gone by since the most commonly used ELISA methods, the INNOTEST assays, for quantification of total tau (T-tau), phosphorylated tau (P-tau), and Aβ42 in CSF were published [63,64,65], showing increased levels of T-tau and P-tau together with decreased Aβ42, a biomarker pattern often called the “Alzheimer’s CSF profile”. Since then, the neuroproteomics field has been rapidly evolving due to improved sample preparation protocols, modification-specific enrichment techniques, proper LC-MS/MS approaches, and PTM-specific data analysis [62]. There has been a significant move from the use of two-dimensional electrophoresis and traditional immunoassays, which might lack sensitivity and specificity, to a larger scale and more in-depth MS-based neuroproteomics studies over the last few years. In MS-based studies, several complex samples of different sources, including brain compartment tissues (post-mortem), brain biopsies, or other body fluids (e.g., blood, plasma, CSF) from patients with different neurological diseases, have been analyzed. Notably, it is interesting to explore other biological sources beyond brain tissue in the biomarker discovery field, as brain tissue implies brain biopsy, which, contrary to brain tumors, is not part of the standard procedure for diagnosing and managing neurodegenerative diseases. However, post-mortem brain tissue samples can serve as a good starting point for identifying new potential biomarkers. Performing untargeted MS proteomics in tissue, followed by confirmation and validation in a body fluid by targeted MS combined with prior enrichment, is a common approach to identify low abundant proteoforms released from the brain. The sample preparation process should account for the heterogeneity of clinical matrices due to the central nervous system’s complex cellular and subcellular architecture and the clinicopathological mechanisms [43]. Postmortem-induced PTM changes such as phosphatase activation can also occur [66], and these mechanisms are likely to strongly modify the relative proportions of some PTMs to others [67]. Therefore, it is important to consider that some PTMs can be lost or degraded during extraction, fixation (if performed), storage, and analysis. In addition, as discussed before, the complexity of the brain, plasma, or CSF matrices can be reduced by using adequate platforms for selective enrichment, mainly if the analysis is focused on PTMs.

Research has demonstrated the possible involvement of aberrant PTMs in disordered or “alternatively folded” proteins that tend to be more vulnerable to aggregation in neurodegenerative diseases [68,69]. Specific PTMs such as phosphorylation, ubiquitination, GlcNAcylation, and nitrosylation can cause alterations in different biological processes such as autophagy and mitophagy, cell-cycle dysregulation, inflammatory response, and mitochondrial dysfunction, which leads to synaptic dysfunction and cognitive disability and ultimately causes neuronal cell death [69]. However, the complete understanding of the role of PTMs in the pathological processes is still impacted by several limitations in the methodology, which is biased toward the peptides of highest intensity and narrowed by accurate site identification of labile PTMs, mostly in complex proteins. To overcome these challenges, analytical methods such as full-length expressed stable isotope-labeled tau (FLEXItau, a mass spectrometry-based assay for tau PTMs quantification) have been recently applied for an unbiased and quantitative analysis of tau PTMs [70,71]. The study identified 95 PTMs on tau isolated from post-mortem brains of AD patients and highlighted the impact of abnormal PTMs on tau aggregation [70].

In summary, considering the poorly understood molecular mechanisms of most brain disorders, the use of neuroproteomics combined with PTM analysis and neuropathology is essential. In that regard, LC-MS/MS approaches offer the opportunity to improve our understanding of the complexity of the underlying biological and molecular pathways that lead to protein deposits in the brain in different neurodegenerative diseases.

## 3. PTM-Proteomic Profiling in Neurodegenerative Diseases by MS

### 3.1. High-Throughput Profiling of Brain PTMs

The measure of the dynamics of the PTM-modified proteome of the brain with high throughput and in-depth coverage MS-based approaches was performed mainly for phosphorylation, ubiquitination, and glycosylation in AD. PTM-profiling the brain tissue using high-throughput MS can substantially provide new insights into the role of aberrant PTM patterns in human disease and identify signatures with potential diagnostic or therapeutic relevance.

#### 3.1.1. Phosphorylation

The precise consequences of protein phosphorylation in the biology and pathogenesis of neurodegenerative diseases, including the mechanisms governing protein misfolding and aggregation, are still unknown. Several studies conducted a global quantitative analysis of the human brain proteome and phosphoproteome in neurodegenerative diseases to elucidate the complexity of these diseases and distinguish them. A phosphoproteome study used a multiplex TMT MS-based approach to quantify the total proteome and phosphoproteome of 27 human post-mortem cortical cases across pathology-free controls, asymptomatic and symptomatic AD patients [31]. They quantified 11,378 unique protein groups and IMAC-enriched 51,736 phosphopeptides. Phosphopeptides were enriched in the IMAC proteome (71%) compared with the total proteome (1.7%). As a measure of quality, they confirmed the presence of increased amyloid-beta and tau levels (the core pathological hallmarks of AD) [31]. Specifically, two peptides of Aβ42 (APP695 region 597–638, peptide 6–16 and peptide 17–28) and tau phosphopeptides (pS191, pT217, pT231, pS262, pS262/T263, and pS289) showed significant increases in asymptomatic AD and AD groups compared with control samples [31]. This comprehensive data on the phosphoproteome of the human brain in AD is valuable for biomarker validation studies.

#### 3.1.2. Ubiquitination

Ubiquitination occurring in proteinopathies is directly or indirectly involved in the impairment of the processing of misfolded material, which is linked with protein aggregation [72,73]. Namely, several neurodegenerative diseases, including AD, FTLD, PD, HD, and CBD, have distinct ubiquitin-positive pathological protein aggregates [73,74,75]. Mapping ubiquitin sites by MS relies on identifying the last two remnant glycine residues (di-Gly, +114.043 Da) of ubiquitin on lysine residues following trypsin digestion [76,77]. Of note, the identification of ubiquitin substrates using MS is challenging due to the low stoichiometry of ubiquitin-modified peptides in complex protein extracts such as brain homogenate [78,79]. Label-free MS-based proteomic analysis identified 4291 unique ubiquitination sites mapping to 1682 unique proteins in human post-mortem brain tissue. By immunoaffinity enrichment of di-Gly isopeptides, over 800 ubiquitination sites were different between AD and control cases [32]. Eighty percent of them are increased in AD, including seven polyubiquitin linkages, consistent with the observed proteolytic stress and high burden of ubiquitinated pathological aggregates. Overall, these findings demonstrate the value of using MS to map ubiquitinated substrates in the human brain of patients with neurodegenerative diseases [32].

#### 3.1.3. Glycosylation

Glycosylation alterations may play critical roles in the disease processes of neurodegenerative diseases [80]. So far, little is known about the role of the altered glycome in the neurodegenerative process. A comprehensive study of the *N*-glycome in the brain dorsolateral prefrontal cortex using MALDI-TOF MS enabled the identification of 141 cortical *N*-glycans that were predominantly sialylated or fucosylated in AD [33]. LC-MS/MS also demonstrated that monosialylated, fucosylated, and bisecting *N*-acetylglucosamine *N*-glycans (often called brain-type *N*-glycans, which are located mainly on some specific CSF proteins [81]) were present in AD brains but not in healthy controls [82]. In both control and AD brains, eleven *N*-glycans had significantly different levels in the hippocampus compared to the cortex [82]. A glycomic study was carried out in CSF samples from eight patients with AD and eight controls, where the *N*-glycans were released, permethylated, and analyzed using an online reverse-phase purification system connected to an LC-LTQ Orbitrap Velos mass spectrometer [83]. A higher expression of bisecting GlcNAc and fucosylated glycans was observed in females with AD (but not males) than in controls, while high mannose structures were underexpressed [83].

Concerning glycoproteomics, a large-scale intact *N*-glycoproteomic approach combining enrichment by hydrophilic interaction chromatography (HILIC) and boronic acid with electron transfer and higher-energy collision dissociation (EThcD) was recently conducted for CSF of patients with AD [84]. A total of 2893 intact *N*-glycopeptides (285 *N*-glycoproteins) were identified, and altered glycosylation patterns in AD (e.g., decreased fucosylation) were detected for several *N*-glycoproteins including alpha-1-antichymotrypsin, ephrin-A3, and carnosinase CN1 [84].

Overall, advances should thus be made to identify modified glycans and glycoproteins involved in the neurodegenerative processes, focusing on brain tissue and biofluids (CSF and serum) to understand their role in this context [80]. These may serve as important targets and may eventually lead to the molecular elucidation of the role of glycosylation in disease mechanism and progression.

### 3.2. Profiling the Main Players in Neurodegeneration

**Figure 1:**
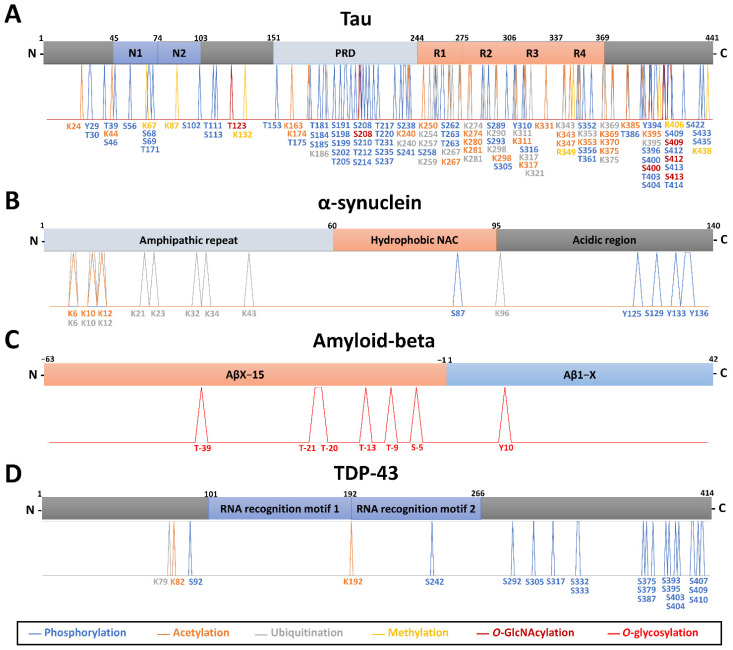
Barcode of the identified post-translational modifications by mass spectrometry on tau (**A**), α-synuclein (**B**), amyloid-beta (**C**), and TDP-43 (**D**) proteins in neurodegenerative diseases. Aβ: amyloid-beta; PRD: proline-rich domain; NAC: non-Abeta component; TDP-43: TAR DNA-binding protein-43.

**Figure 2:**
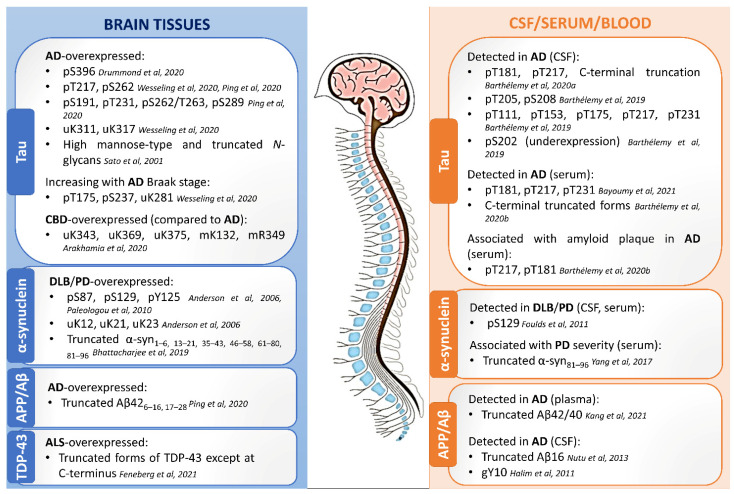
Relevant PTMs identified by mass spectrometry as being preferentially modified in the brain tissues, CSF, and blood/serum in neurodegenerative diseases, with potential use as biomarkers. A: acetylation; Aβ: amyloid-beta; AD: Alzheimer’s disease; APP: amyloid-beta precursor; CBD: corticobasal degeneration; CSF: cerebrospinal fluid; DLB: dementia with Lewy bodies; g: glycosylation; p: phosphorylation; PD: Parkinson’s disease; TDP-43: TAR DNA-binding protein-43; u: ubiquitination [24,31,34,67,70,85,86,87,88,89,90,91,92,93,94,95,96,97,98] (These references are cited in the figure).

Profiling the PTM-modified proteins from the neuropathological hallmark aggregates, including tau, α-synuclein, amyloid-beta, and TDP-43, has been performed in different biological milieus using MS. A summary of the main findings of PTM localization sites identified by MS is found in Figure 1. An overview of the relevant PTMs identified by MS with potential for diagnosis or detection of disease progression in neurodegenerative diseases is illustrated in Figure 2.

#### 3.2.1. Tau Protein

Phosphorylation of the microtubule-associated protein tau is one of the most important PTM for axonal stabilization and regulation [99]. Still, abnormally phosphorylated tau (P-tau, phosphorylated at non-physiological sites) appears to lead to neurofibrillary tangles (NFTs) that no longer stabilize microtubules [100]. Numerous research, including large-scale studies [31], have consistently reported that tau levels (total tau and hyperphosphorylated tau) are prominently increased in the AD brain tissues [67,101], CSF [102], and plasma [85]. Total tau is linked to the severity of neurodegeneration, whereas phosphorylated tau reflects specific AD pathological changes in the neurofibrillary system [103]. Tau hyperphosphorylation seems to be required but is insufficient to induce tau aggregation; other less investigated tau PTMs are certainly involved [104,105]. For example, several other PTMs, including ubiquitination and methylation, influence tau filament structure by contributing to the structural diversity of tauopathy strains [86] and may play an important role in tau localization and protein–protein interactions [106]. Analysis and quantification of tau PTMs in the brain and biofluids by MS is mainly focused on AD and CBD and is still missing for the other neurodegenerative diseases.

**Brain tissues.** Brain soluble tau phosphorylation sites are mainly localized at the C-terminus, at proline-rich mid-domain, and a cluster on the N-terminal projection domain [67]. Tau’s longest isoform (2N4R, 441 aa) has 85 potential phosphorylation sites, and almost 20 residues were found to undergo phosphorylation in the healthy brain [107]. In brain tissues from seven patients with advanced sporadic AD, LC-MS identified 542 proteins in NFTs and tau in all seven cases [34]. Tau was phosphorylated on 23 different residues, and the most abundant tau phosphosite was pS396 [34]. Other studies published before the year 2000 report various tau phosphorylation sites in AD after enrichment with monoclonal antibodies against tau and MS analysis [107,108,109]. Recently, Barthélemy et al. [67] increased the number of known tau phosphorylated sites to 29, identified pS404 as the most abundant species in the brain, and 12 of these phosphopeptides were common to CSF in AD by an in-depth targeted MS (PRM) analysis, independently of tau concentration. Phosphorylation of some particular sites was exacerbated (or specifically detected in AD) compared to controls, supporting the hypothesis that tau phosphorylation could be a physiological process amplified by AD pathology [67]. Tau phosphopeptides pS191, pT217, pT231, pS262, pS262/T263, and pS289 showed a significant increase in AD compared with control samples [31]. Using an exploratory IP-MS approach, tau phosphorylation on brain soluble fraction was shown to reflect CSF, with pT181, pT217, and pT231 among the most prominent species identified in AD [110]. A doubly-phosphorylated tryptic peptide (pT231 + pS235) specific of AD brain was identified in higher amounts than the monophosphorylated (pT231) counterpart [110]. Using sarkosyl-insoluble samples, Wesseling et al. [70] performed a quantitative and qualitative tau protein profiling (FLEXITau and Q-Exactive MS) of 29 AD patients and 28 matched control individuals. The study demonstrated that the abundance of insoluble tau is higher in AD than in healthy controls, with pathogenic tau aggregates predominately composed of the 0N, 1N, and 4R isoforms. The most relevant phosphosites specific to AD were pS199, pS202, and pT205 [70]. The total number of phosphorylated residues identified by all methods in normal or AD/CBD human brain tissue stands at 56, representing well over half of all hydroxyl amino acids in tau [70] (Figure 1A).

Tau acetylation (identified on 21 lysines) directly contributes to the accumulation of phosphorylated tau, affecting tau turnover in CBD and AD [70,86,111] (Figure 1A). Acetylation at K281, K331, K343, and K353 of tau fibrils from CBD patients’ brains and at K298, K311, K331, K343, K353, and K369 from AD patients was described thanks to Orbitrap MS [86]. Within the tau proline-rich domain, K163, K174, and/or, K180 have been reported as acetylation sites by immunoprecipitation and MALDI-TOF MS, with occupancy detectable in a normal brain and increasing with Braak stage in AD brain [112].

Tau has the highest number of ubiquitination sites (17 sites) per any protein in AD, as identified by Orbitrap MS [32,70,101]. Tau is almost exclusively ubiquitinated and acetylated in the tandem repeats R1-R4 and K369-E380 of sarkosyl-insoluble fractions from CBD and AD post-mortem tissue [86]. Within these regions, ubiquitination can also occur at different sites of tau for AD (K254, K259, K267, K311, K317, K321) and CBD (K254, K343, K369, K375) [86] (Figure 1A). Recently, a structure-based model in which specific ubiquitination of tau influences the resulting filamentary structure was built by combining results from LC-MS analysis and cryo-electron microscopy observations using CBD patients’ brains [86].

Tau methylation is a relatively recent discovery with several lysine residues being methylated in AD patient brains (4 sites: K67, K87, R406, K438) [70,113] and CBD (2 sites: K132, R349) [86], as shown by Orbitrap MS (Figure 1A). Interestingly, tau methylation appeared distributed among at least 11 sites, in the form of mono- and dimethyl lysine residues, primarily focused on the microtubule-binding repeat region, in four cognitively normal human brains using Orbitrap MS [114].

High mannose-type sugar chains and truncated *N*-glycans were found on tau in addition to a small amount of sialylated bi- and tri-antennary sugar chains. Truncated glycans were found richer in AD paired helical filaments (PHF)-tau than in AD cytosolic phosphorylated tau, which has been suspected of promoting the assembly and/or the stability of the pathological fibrils in AD [87]. In addition, tau can be glycosylated by *O*-GlcNAc (Figure 1A), which is inversely proportional to the amount of phosphorylation [115,116] and responsible for slowing down neurodegeneration and preventing aggregation [117]. Confirmation of these observations requires further investigation of the relative abundance of tau PTMs and isoforms in the brain, then in CSF, and ideally plasma, for biomarker discovery.

**CSF.** Quantitative high-resolution MS/MS strategies, including PRM, have given new insights into tau metabolism and truncation [118,119]. Specifically, these highlight differences in the relative abundance of PTMs between the brain and CSF tau of AD patients, notably, differences in truncations of the C-terminus [119] and opposite trends in phosphorylation rate depending on the sites. A recent publication also identified site-specific phosphorylation changes in CSF along with the AD progression, particularly a reversal of pT181 at the onset of cognitive decline, which suggests sequestration in the brain of specific tau species [88]. These recent observations raised the question of the relationship between CSF and brain tau. In the CSF, detection of soluble pT181, pT217, and truncated tau forms (e.g., Tau368) have been investigated for differential diagnosis and distinguish AD from other dementias using an Orbitrap MS [24,88]. The release of pT217 from the brain to CSF was linked to increased phosphorylation in AD. CSF pT217 was more accurate in detecting the presence of amyloid plaques (identified by PET) than other sites, such as pT181 [24,120] and pT205 [88]. Remarkably, increased CSF levels of pT217 are closely related to amyloid plaques at the asymptomatic stage [121]. Phosphorylation occupancy on pT217 is also lower intracellularly in the brain than extracellularly in CSF [67]. Phosphorylation on pT205 and pS208 was detected in the CSF but not in the brain from healthy controls [67]. Interestingly, the benefit of combining tau and α-synuclein for differentiation of DLB, AD, and controls was recently suggested [122].

**Blood/Serum.** Brain and CSF proteins are transferred to the blood through the blood-brain barrier, arachnoid granulations, and the glymphatic system [123]. Different studies have demonstrated that disease-associated protein alterations in the brain can be detected in the blood. A similar tau C-terminal truncation pattern in plasma compared to CSF was reported, with 15 tau peptides from residues 6–254 being detected, including 0N, 1N, 2N, and 3R-specific peptides using Orbitrap MS [85,118,119,124]. An inferred abundance of 0N/1N/2N peptides indicated similar contributions to previous reports in the brain and CSF (∼5/5/1) [119]. pT217 and pT181 in plasma were highly specific for amyloid plaque pathology [85]. However, there is strong evidence of a non-brain peripheral contribution of tau in plasma, with a different phosphorylation profile than CSF [89]. Findings support blood phosphorylated tau isoforms (pT181, pT217, and pT231) as potentially helpful in detecting AD pathology, staging the disease, and diagnosis [89].

#### 3.2.2. α-Synuclein

α-synuclein is widely distributed in the CNS and is involved in the packaging, trafficking of vesicles, and regulation of synaptic plasticity [125]. For reasons not fully comprehended, α-synuclein is prone to misfolding and forming fibrillar and aggregated forms within Lewy bodies and Lewy neurites-a typical pathological hallmark of PD and other synucleinopathies [126]. The molecular factors triggering α-synuclein aggregation and Lewy bodies formation remain unknown. Changes in α-synuclein phosphorylation could represent a response to biochemical events associated with PD pathogenesis [127].

**Brain tissues.** α-synuclein within Lewy bodies is phosphorylated at S87, S129, or Y125 as deduced from MS data in PD [90,128]. Phosphorylated α-synuclein at S129 accounts for more than 90% of α-synuclein found in Lewy bodies [129]. In contrast, only 4% or less of total α-synuclein is phosphorylated at this residue in the normal brain [91,128]. This suggests that the accumulation of S129-phosphorylated α-synuclein is somehow related to the formation of Lewy bodies and dopaminergic neurodegeneration in PD [90,128]. Ubiquitination (K12, K21, and K23, in phosphorylated α-synuclein forms) is only detected in Lewy bodies [91]. Together with truncation, these ubiquitinated and phosphorylated forms are present in the detergent-insoluble fraction of the familial PD brain (synuclein A53T mutation) [91]. Several other α-synuclein proteoforms, including accumulation of truncated forms at C-terminal (Ac-α-syn_1–119_) and N-terminal (α-syn_71–140_, α-syn_68–140_, α-syn_66–140_, α-syn_65–140_), mainly in the cingulate cortex, were described in PD [130]. The levels of α-synuclein forms in Lewy body-enriched α-synuclein fraction (α-syn_1–6_, α-syn_13–21_, α-syn_35–43_, α-syn_46–58_, α-syn_61–80_, and α-syn_81–96_) were significantly increased in the PD cingulate region compared to controls [92]. In addition, brain-derived α-synuclein is mainly N-terminally acetylated in Lewy body-enriched PD brain tissue fractions, as characterized by intact protein LC-Orbitrap MS [92] (Figure 1B). Although phosphorylation, acetylation, ubiquitination, and truncation may play an important role in α-synuclein biology, our understanding of the precise effects of these modifications in the biology and pathophysiology of neurodegenerative diseases is still partial. Of note, deposits of α-synuclein and S129-phosphorylated α-synuclein in the brain could be detected in people with non-neurodegenerative disorders [22].

**CSF/Blood/Serum.** Monomeric, oligomeric, and post-translationally modified α-synuclein can be detected in body fluids such as CSF, plasma, and red blood cells [131,132,133]. When analyzing α-synuclein as a biomarker, the high concentration in red blood cells should be considered and requires monitoring of blood contamination in CSF [122]. Mass spectrometry using MRM mode showed CSF synuclein concentrations 550% superior to ELISA but similar levels in serum of subjects without neurodegenerative diseases [133]. This difference could indicate the presence of different α-synuclein species in serum and CSF. CSF synucleins (alpha, beta, and gamma) were detected as being increased by MRM in AD and CJD, but no alteration was detected for synucleinopathies (PD, PD dementia (PDD), DLB) [133]. Moreover, α-synuclein peptides α-syn_61–80_ and α-syn_81–96_ from the non-Abeta component (NAC) region have a 38 and 40% lower concentration in CSF than α-syn_24–32_ from the N-terminal region [133]. In 2020, the same group found an increased β-synuclein in patients with mild cognitive impairment and CJD, but not for PD [134]. pS129 and ubiquitination (at multiple sites) have been detected in the CSF and plasma of PD, MSA, and DLB cases [93]. In red blood cells, Pero-Gascon et al. [135] detected N-acetylated α-syn as the main proteoform in healthy individuals and some PD patients (stage III and IV). Yang et al., 2017 found that α-syn_81__–__96_, TVEGAGSIAAATGFVK, was highly correlated with disease severity and allowed the tracking of the PD progression [94].

#### 3.2.3. Amyloid Precursor Protein (APP) and Amyloid-Beta Peptides

The function of amyloid precursor protein (APP) in the body is unknown; however, evidence suggests its role in synaptic formation and function, neuronal outgrowth, protein trafficking, signal transduction, and intracellular calcium homeostasis. In pathological conditions, the proteolytic processing of APP by the β and γ secretase, which cleaves the protein at specific sites, generates Aβ peptide fragments with variable lengths of 37–43 amino acids. Among them, Aβ42 peptides are more prone to aggregation [136]. In the brain, Aβ42_6–16_ and Aβ42_17–28_ (APP695 region 597–638) showed significant increases in asymptomatic AD and AD groups compared with control samples [31]. The Aβ42/Aβ40 ratio in plasma was measured with LC-MS in AD and mild cognitive impairment patients to see its correlation to amyloid PET status and was shown to be a good predictor of Aβ PET positivity [95]. Using combined immunoprecipitation and HR-MS techniques, it was shown that the relative levels of Aβ16 in AD compared to controls are increased in CSF [96]. It was identified in AD 37 APP/Aβ glycopeptides with sialylated core-1 like *O*-glycans attached to Thr(−39, −21, −20, and −13) in a series of APP/AβX–15 glycopeptides, where X was −63, −57, −52, and −45, concerning Asp1 of the Aβ sequence [97]. An increase in Y10 *O*-glycosylated Aβ peptides linked to (Neu5Ac)_1–2_Hex(Neu5Ac)HexNAc-O-structures was observed in CSF in six AD patients compared to seven non-AD patients [97]. Short Aβ isoforms (N-terminal −3, 1, 4, 5; C-terminal 15, 16, 17, 18, 19, 20) were detected with *O*-glycosylation at Y10 of Aβ [97,137] (Figure 1C). Despite this, further investigations are warranted to address the significance of glycosylation in APP proteolysis and its consequences on amyloid deposition.

#### 3.2.4. TAR DNA-Binding Protein (TDP-43)

TDP-43 is a highly conserved nuclear factor encoded by the *TARDBP* gene to regulate transcription and alternative splicing [138]. During a pathological context such as ALS and FTLD, TDP-43 translocates to the cytoplasm with deletions at the C-terminal glycine-rich region (35 and 17–27 kDa) [139,140]. In the human ALS brain, TDP-43 was characterized by LC-LTQ-MS and demonstrated 17 phosphorylation sites, K79 ubiquitination, and K82 acetylation [138] (Figure 1D). More recently, Feneberg et al. [98] used highly sensitive MS with PRM to quantify the abnormal enrichment of C-terminal TDP-43 fragments in ALS brain insoluble fractions. A C/N-terminus ratio >1.5 discriminated ALS from controls with 100% sensitivity and specificity in this study. Despite TDP-43 being identified in CSF [98,141], no studies were performed using LC-MS.

## 4. The Next Step in Neuroproteomics

Although it has enjoyed remarkable recent success stories, proteomics still faces substantial technical challenges. Proteomics data processing and analysis is a multistep process that requires a lot of expertise. Multiple stages are necessary for the consistent analysis of LC-MS and LC-MS/MS data. Adequate sample preparation, reducing complexity, and enriching lower abundance components while depleting the most abundant can help solve the analysis challenges. Sample enrichment using immunoprecipitation of the target protein, fractionation, or PTM-specific enrichment methods (chemical and biochemical) are now pretty well established [142]. Various offline fractionation methods have been employed to enhance the depth of the proteome by improving the detection of low abundance peptides before LC-MS/MS analysis. Methods including two-dimensional gel electrophoresis [143], strong cation exchange (SCX), electrostatic repulsion-hydrophilic interaction chromatography (ERLIC) [30], and high-pH reversed-phase chromatography [144] are used to increase peptide identification by separating peptides in an orthogonal dimension. Unfortunately, sample processing is still the main bottleneck for many extensive proteomics studies because it is a complex multiple-step process. There is no standard best approach for sample processing, and optimizing this step is detrimental to the ability to study the proteoform(s) of interest effectively.

Additionally, state-of-the-art mass spectrometry instrumentation and extensive automatized high-confident data processing and analysis are required. Label-free quantification, super-SILAC, and chemical labels can be employed for large-scale quantitative discovery. Modified peptides that may serve as biomarkers can be validated with larger cohorts using targeted MS methods such as MRM or PRM. Finally, with MS instrumentation improving, the bottleneck shifts towards confirming PTM sites and validating their function using new experimental and computational strategies. The exploitation of this technology resulted in significantly enhanced protein sequence coverage, the discovery of undescribed modifications, and the parallel analysis of different types of modification sites. These studies also generally characterized only a limited number of PTM types, with the most robust emphasis on phosphorylation and little focus on less common PTMs such as acetylation or glycosylation.

Furthermore, most MS-based experiments employed DDA, a data collection mode that relies on the “detectability” of the peptide species of interest, biasing the analysis towards peptides of the highest intensity. This particularly handicaps the identification of PTMs, as the modified species can be present in very low stoichiometries and/or exhibit lower detectability compared to their unmodified counterpart [145]. In addition, studies have now tried to understand the impact of PTM crosstalk [54]. Notably, the combination of PTMs, rather than individual PTMs, define protein function and are involved in the pathological mechanisms underlying neurodegenerative diseases. However, the current limitations in large-scale PTM combinatorial analyses render developing adequate clinical biomarker assays that target peptides with different modifications extremely challenging. This might be facilitated in the future with the continuous development of next-generation mass spectrometry workflows, i.e., top-down or native MS [146,147].

**Figure 3:**
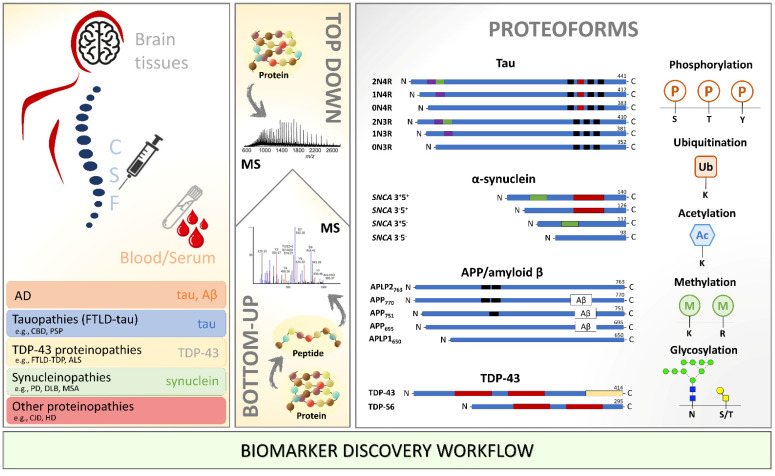
Biomarker discovery workflow using mass spectrometry for identification and characterization of proteoforms in the different biological milieu of patients with neurodegenerative diseases. Amyloid β: amyloid-beta; APP: amyloid-beta precursor; ALS: amyotrophic lateral sclerosis; CBD: corticobasal degeneration; CJD: Creutzfeldt–Jakob disease; CSF: cerebrospinal fluid; DLB: dementia with Lewy bodies; FTLD: frontotemporal lobar degeneration; HD: Huntington’s disease; MS: mass spectrometry; MSA: multiple system atrophy; PD: Parkinson’s disease; PSP: progressive supranuclear palsy; SNCA: synuclein alpha; TDP-43: TAR DNA-binding protein 43.

There is optimistic thinking about what the next few decades in the neuroscience field will bring. With the neurodegenerative cases increasing as the global population ages, a significant effort will have to be made to improve the complete integration of robust but sensitive MS-based proteomics approaches into biomarker discovery facilities (Figure 3) and, ultimately, into clinical settings. First, however, it is crucial to understand (1) how PTMs regulate the cellular events; (2) how to rank by importance the several PTMs occurring on a single protein; and (3) what are the switch on-and-off responses and crosstalks of PTMs to pathological events, considering a particular pathological tissue or biofluid in a certain disease stage. The aim is to identify unique pathological signatures and biomarkers that can aid in understanding and monitoring the different processes involved in disease development and progression. For this, it is vital to limit inconsistencies across clinical studies by studying well-classified patient material and large patient cohorts and standardizing methodologies and protocols. Therefore, the field must now focus on large-scale multicentric MS-based translational studies integrating molecular signatures with pathological findings to diagnose and stratify neurodegenerative diseases. Integrative proteomic and genomic/transcriptomic analyses (in neuroproteogenomics settings) hold great promise for understanding the role of the PTMome in neurodegenerative diseases. For example, a multi-omics data fusion from 177 studies and more than one million patients with AD, PD, HD, and ALS has recently shed light on different biological processes between these pathologies [148]. However, PTMome information was not considered in this integrative study.

Interestingly, by including metabolomics approaches, it is possible to understand the PTM relative changes by monitoring the precursors of protein PTMs (e.g., acetyl-CoA for acetylation, ATP for phosphorylation, and S-adenosylmethionine for methylation) [149]. Therefore, analysis of PTMs in multi-omics studies will almost certainly reveal novel and even unexpected molecular signatures. This will allow for a better understanding of the molecular pathways underlying the development of neurodegenerative diseases, thereby facilitating the decision-making process and precision medicine settings. Although these integrative approaches are promising, there is still a long way to go to routinely incorporate omics data into clinical decisions for personalized interventions.

## 5. Concluding Remarks

High-throughput proteomics and PTM-omics studies using MS have been used in recent years to investigate neurodegenerative diseases. Great potential is underlying specific pathological PTM-signatures for clinical applications as biomarkers in neurodegenerative diseases. This is the case of diverse modified peptides from the major players in neurodegeneration processes such as tau, α-synuclein, APP/amyloid-beta, or TDP-43. Tau protein remains the most well-studied protein using MS. Several phosphorylated, acetylated, ubiquitinated, methylated, or glycosylated tau peptides are accumulated in the brain of AD and CBD or associated with a higher AD Braak stage. In addition, phosphorylated and C-terminal truncated forms of tau can be detected in CSF and serum in AD. For α-synuclein, studies focus more on phosphorylated, ubiquitinated, and truncated proteoforms that could be overexpressed specifically in tissues, CSF, and serum from DLB or PD patients. To the best of our knowledge, TDP-43 truncated forms were only studied in ALS brain tissues, and amyloid-beta was only analyzed in AD CSF. We believe that major breakthroughs can occur in detecting and characterizing PTMs less studied in the field using HR/MS. Due to the lack of data on non-AD neurodegenerative diseases, we also envisage that these will undoubtedly be the focus of neuroproteomics studies.

## Data Availability

Not applicable.
